# Top-Down Design Method of a Time Domain Accelerometer with Adjustable Resolution

**DOI:** 10.3390/mi15050635

**Published:** 2024-05-09

**Authors:** Enfu Li, Jiaying Jian

**Affiliations:** 1 School of Engineering, Huzhou University, Huzhou 313000, China; 2 School of Electronic Information and Engineering, Xi’an Technological University, Xi’an 710021, China

**Keywords:** MEMS, inertial sensor, accelerometer, time domain measurement, design methodology, adjustable acceleration resolution

## Abstract

A top-down design methodology and implementation of a time domain sensor is presented in this paper. The acceleration resolution of the time domain sensor is equal to the time-measurement accuracy divided by the sensor sensitivity. Combined with the sensitivity formula, the acceleration resolution is proportional to the vibration amplitude, the time-measurement accuracy, and the third power of the resonant frequency. According to the available time-measurement accuracy and the desired acceleration resolution, the parameters including the vibration amplitude and the resonant frequency were theoretically calculated. The geometrical configuration of the time domain sensor device was designed based on the calculated parameters. Then, the designed device was fabricated based on a standard silicon-on-insulator process and a matched interface circuit was developed for the fabricated device. Experimental results demonstrated that the design methodology is effective and feasible. Moreover, the implemented sensor works well. In addition, the acceleration resolution can be tuned by adjusting the time-measurement accuracy and the vibration amplitude. All the reported results of this work can be expanded to other time domain inertial sensors, e.g., a gyroscope or tilt sensor.

## 1. Introduction

Microelectromechanical system (MEMS) accelerometers have gained widespread use in the consumer electronics and automobile industries and geophysical exploration due to their advantages of low cost, small size, light weight, and low power (CSWaP) [1,2,3,4,5]. The first MEMS accelerometer, a piezoresistive sensor, was developed by L. M. Roylance and J. B. Angel at Stanford University in 1979 [6]. Since then, researchers have developed various types of accelerometers based on different sensing mechanisms, including piezoelectric [7], resonant [8], mode-localized [9,10], tunneling [11], capacitive [12], etc. Piezoresistive accelerometers offer a measuring technique (widely using piezoresistive bridges) and exhibit good linearity and a wide dynamic response range. However, these sensors are sensitive to temperature variations, necessitating the implementation of temperature-compensation mechanisms [13]. Piezoelectric accelerometers demonstrate good linearity and a wide dynamic response range, but are not suitable for low-frequency or static measurements [14]. Resonant accelerometers provide excellent stability, high accuracy, and strong resistance to interference. However, they have a limited frequency response range [15,16]. Mode-localized accelerometers utilize the energy confinement of mode localization to enhance accuracy, with relative sensitivity to amplitude ratio 302 times higher than the resonance frequency shift [17]. Despite this improvement in accuracy, mode-localized accelerometers suffer from poor stability in amplitude ratio output, limiting their use in high-accuracy fields. Among these sensing mechanisms, capacitive accelerometers have emerged as a research hotspot and have gained favor in the commercial industry [18,19,20]. The state-of-the-art resolution of capacitive sensors is currently around 0.44 µg [21] according to the published literature. However, further improvements in the accuracy of capacitive sensors are challenging [21,22].

A time domain acceleration-sensing mechanism that converts acceleration perturbation into changes in time intervals was theoretically proposed by Space and Naval Warfare Systems Center Pacific (SSC Pacific). This sensing mechanism theoretically has the great potential to achieve a detection limit of ~10^−13^ g [23,24] because the state-of-the-art time accuracy arrives at the level of 10^−18^ s [25]. A theoretical implementation method based on tunneling electrodes was also presented by SSC Pacific [26]. Based on the implementation method, some theoretical works have been reported, mainly focusing on the apparatus and appliance of in-plane inertial devices [27], the intelligent polynomial curve fitting method for inertial devices [28], modeling gyroscope devices, and the angular random walk estimation method for gyroscopes [29]. However, none of the time sensors were developed, due to the methods being hard to implement. As a result, the theoretical results have not been experimentally verified.

Since then, the literature with respect to time domain sensors has been seldom published except by our research group. In our previous works, several novel characteristics of time domain accelerometers have been identified and verified utilizing a self-developed sensor based on a standard silicon-on-insulator process. First, a virtual time domain accelerometer array using a single device was built [30]. Multiple acceleration measurements could be simultaneously performed using the built virtual time domain sensor array. The accuracy was improved by combining all the measurements. Second, the time domain sensor has the ability to measure acceleration perturbation during the process of attenuation vibration [24]. Third, two sensitivities exist in the time domain sensors, which can be enhanced by decreasing the amplitude and frequency [23].

However, the design method of time domain sensors and their new characteristics have seldom been reported in the published literature to data. In this paper, a top-down design methodology for a time domain sensor device is proposed. Based on this methodology, the structural topology of a self-developed sensor device was designed step by step and the parameters were optimized. In addition, a corresponding detection method was developed. Finally, utilizing the developed sensor device and the detection method, the time domain sensor with adjustable resolution was experimentally verified.

The rest of this paper is organized as follows. In Section 2, the adjustable resolution of the time domain accelerometer is theoretically analyzed. The design and fabrication of the time domain accelerometer device is presented in Section 3. In Section 4, the detection method of the developed time domain sensor is described. Measurement results and discussion are presented in Section 5. The final conclusions are summarized in Section 6.

## 2. Adjustable Resolution of Time Domain Accelerometer

The acceleration resolution caused by the time-measurement accuracy is equal to the ratio of the time-measurement accuracy Δt divided by the sensitivity S of the time domain sensor. Therefore, the acceleration resolution can be expressed by

(1) $$\Delta a=\frac{\Delta t}{S}$$

where the sensitivity can be expressed by [23]

(2) $$S\propto \frac{2}{A\omega^{3}}$$

where A and ω denote the vibration amplitude and resonant frequency of the time domain sensor, respectively. Submitting Equation (2) into Equation (1), the acceleration resolution can be rewritten as:

(3) $$\Delta a\propto A\omega^{3}\Delta t$$

It can be seen from Equation (3) that the acceleration resolution is proportional to the vibration amplitude, the third power of the resonant frequency, and the time-measurement accuracy. Therefore, Equation (3) is a theoretical foundation for designing a time domain sensor with adjustable resolution.

## 3. Design and Fabrication of a Time Domain Accelerometer Device with Adjustable Resolution

### 3.1. Design of a Time Domain Accelerometer Device

#### 3.1.1. Overall Design

The model of the time domain accelerometer is equivalent to a forced vibration of a mass–spring–damper single-freedom system, where the mass denotes inertial proof mass, the spring denotes elastic beam, and the damper refers to energy loss during the processing of microstructure motion. In addition, a set of (two) displacement reference points (DRPs) is necessary for implementing a time domain sensor, where the DRPs are used for generating the trigger events when the proof mass passes through them. Then, the acceleration is calculated through measuring the time intervals between the corresponding trigger events.

**Figure 1:**
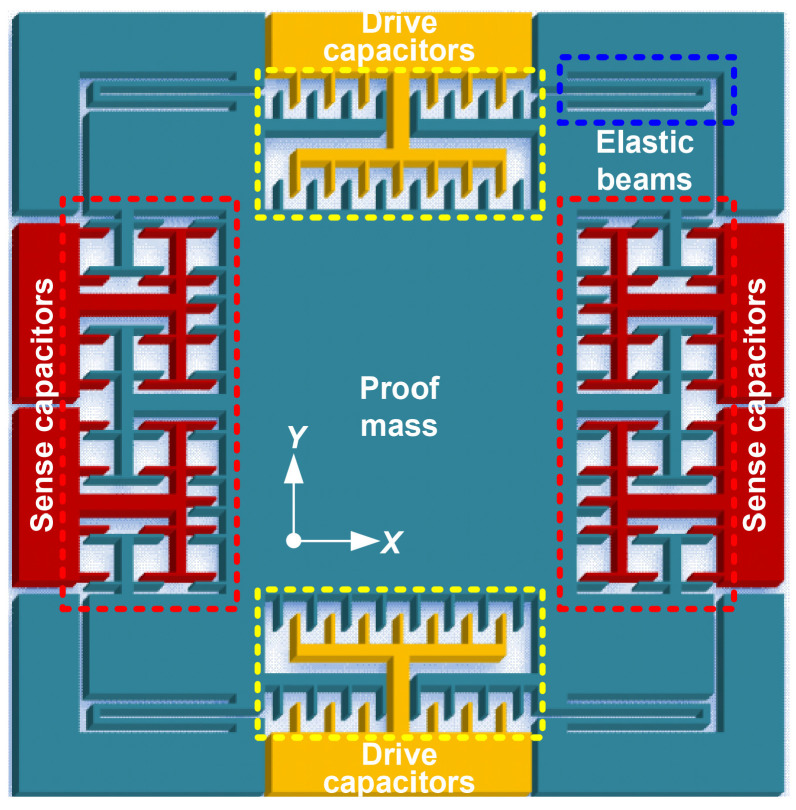
Schematic diagram of the proposed time domain accelerometer device.

According to the model of the time domain sensor, the proposed senor device is shown in Figure 1. The harmonically oscillating proof mass is suspended by the four symmetrical elastic beams and is driven by an electrostatic force generated via area-varying drive capacitors. In addition, the amplitude of the proof mass can be adjusted by changing the drive voltage on the drive capacitors. The gap-varying sense capacitors, combined with a corresponding capacitance-to-voltage (C–V) interface circuit and a self-developed data post-processing algorithm, are utilized for defining the DRPs, generating the trigger events, measuring the time intervals, and further calculating applied acceleration perturbation.

**Figure 2:**
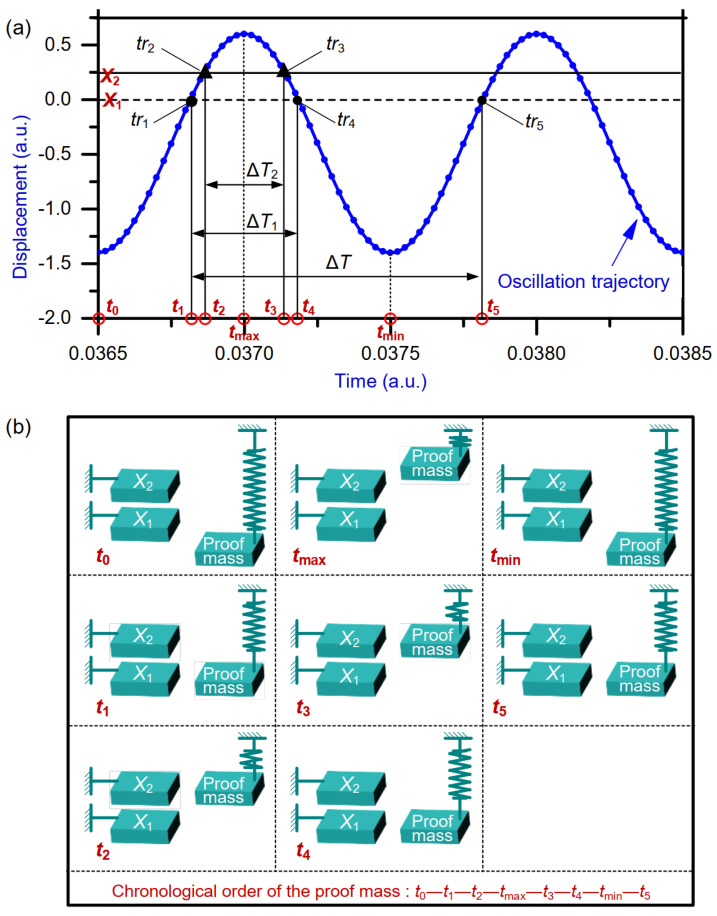
Schematic diagram: (**a**) time interval measurements, (**b**) position relationships between the proof mass and the DRPs at different times.

When an inertial acceleration perturbation is applied in the *Y*-axis positive direction, the proof mass moves in the negative direction along the *Y* axis (Figure 1). The oscillation trajectory of the time domain sensor with acceleration perturbation is shown in Figure 2a. The data points (represented by the blue dots) on the oscillation trajectory denote the proof mass displacements relative to the rest position. The position relationships between the proof mass and the two preset DRPs at different times are illustrated in Figure 2b. The proof mass starts with the lowest position at time $t_{0}$ and then moves up to the DRP $X_{1}$ at time $t_{1}$ and the DRP $X_{2}$ at time $t_{2}$. It reaches the highest position at time $t_{\max}$ and then moves down to the DRP $X_{2}$ at time $t_{3}$ and the DRP $X_{1}$ at time $t_{4}$. The proof mass returns to its original position at time $t_{\min}$. After that, it moves to the DRP $X_{1}$ again at time $t_{5}$ and continues this periodic repetitive motion. In a period, the time intervals $\Delta T_{1}$, $\Delta T_{2}$, and ΔT can be measured by the obtained five times $t_{1}$, $t_{2}$, $t_{3}$, $t_{4}$, and $t_{5}$. Finally, the acceleration can be calculated from the measured $\Delta T_{1}$, $\Delta T_{2}$, and ΔT via the function $a=f\left(\Delta T_{1},\Delta T_{2},\Delta T\right)$.

In this work, for acquiring these five time measurements and finally calculating the acceleration, the displacement of the proof mass is converted into measurable voltage. Because the movable electrodes of the sense capacitors are rigidly connected with the proof mass, they experience the same displacement as the proof mass (Figure 1). Therefore, the displacement of the proof mass caused by the acceleration perturbation is first converted into the change in capacitance. Then, the change in capacitance is converted into that in voltage by the aforementioned C–V interface circuit. As a result, a function relationship between the displacement and voltage is built. The displacement is represented by the output voltage. Correspondingly, the DRPs can be represented by the VRPs.

The National Instruments serial data acquisition (DAQ) system [31] is used to sample voltage signal from the C–V interface circuit. The sampling rate can be up to 2.5 million samples per second (MSPS). Correspondingly, the highest time-measurement accuracy (reciprocal of MSPS) is 400 ns. Thereinto, the time-measurement accuracy is adjustable through varying the sampling rate. Based on our experience from standard silicon-on-insulator processes, the resonance frequency of the developed sensor device should be larger than or equal to 1.2 kHz, in case the proof adheres to the handle layer. The desired acceleration resolution is ~5 mg. According to Equation (3), the maximum vibration amplitude of the time domain sensor is ~584 nm. The size of the device was designed to be 3.5 mm × 4 mm and the sensing mass 1.8 × 10^−7^ kg. The overall parameters are listed in Table 1. Based on the parameters, the elastic beam, driving structure, and sensor structure were designed as follows.

It should be noted that if some parameters of Equation (3) are constrained, the remaining ones can be calculated and optimized. Then, the corresponding parameters of the sensor device and detection circuit can be calculated and designed.

#### 3.1.2. Elastic Beam

**Figure 3:**
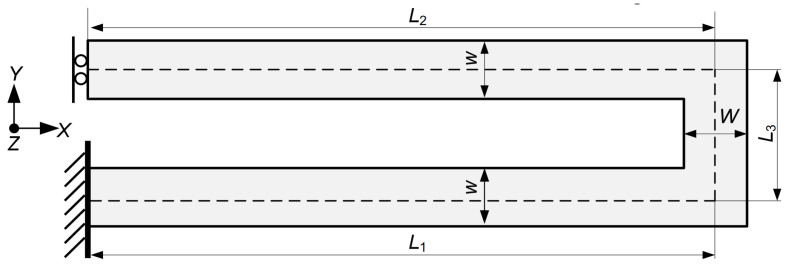
U-shaped elastic beam of this work.

According to the resonant frequency f of 1.2 kHz and the sensing mass m of ~1.8 × 10^−7^ kg (Table 1), the stiffness coefficient K of the elastic beam is ~10 N/m ($f=\sqrt{K/m}/2\pi$). There are various types of elastic beams, including folded elastic beam, L-shaped elastic beam, U-shaped elastic beam, serpentine elastic beam, and so on. Among them, the U-shaped elastic beam (Figure 3) was chosen due to the advantages of low stiffness coefficient in detection mode and good residual stress release [32].

The stiffness coefficients of the U-shaped elastic beam in X, Y, and Z direction, i.e., $K_{x}$, $K_{y}$, and $K_{z}$ can be expressed via Equation (4) [32]:

(4) $$K_{x}=\frac{EtwW^{3}}{2L_{1}W^{3}+L_{3}^{3}w},\;K_{y}=\frac{Etw^{3}W}{L_{3}w^{3}+2L_{1}^{3}W},\;K_{z}=\frac{Et^{3}wW}{2{L_{1}}^{3}W+L_{3}^{3}w}$$

**Figure 4:**
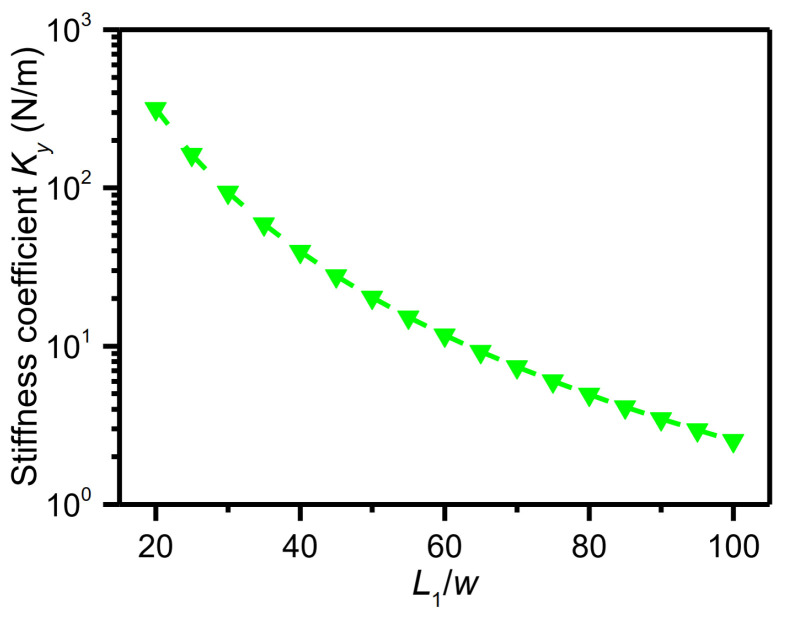
Dependence of the stiffness coefficient in the Y direction on the aspect ratio $L_{1}/w$.

In Equation (4), w and W denote the width of the beam while $L_{1}$, $L_{2}$, and $L_{3}$ denote the length of the beam, as shown in Figure 3. t denotes the height of the beam in the Z direction. Some parameters of the U-shaped elastic beam are constrained: W=30 μm; $L_{3}=30$ μm; $L_{1}/w=L_{2}/w=20\sim 100$. According to Equation (4), the dependence of the stiffness coefficient in the Y direction on the aspect ratio $L_{1}/w$ is displayed in Figure 4. As can be seen from Figure 4, the stiffness coefficient is 10 N/m when the aspect ratio is ~65. In this work, the aspect ratio was taken as 70 for convenient design. For reducing the lateral interference, the ratio of the stiffness coefficients between the non-sensitive direction and sensitive direction should be kept to at least 10-fold. According to Equation (4), when the beam width w is equal to 10 μm, the ratio of the stiffness coefficients of the X direction to the Y direction is ~5000 and the ratio of the stiffness coefficients of the Z direction to the Y direction is ~10. The geometrical parameters of the designed U-shaped elastic beam are listed in Table 2.

#### 3.1.3. Electrostatic Driving Structure

**Figure 5:**
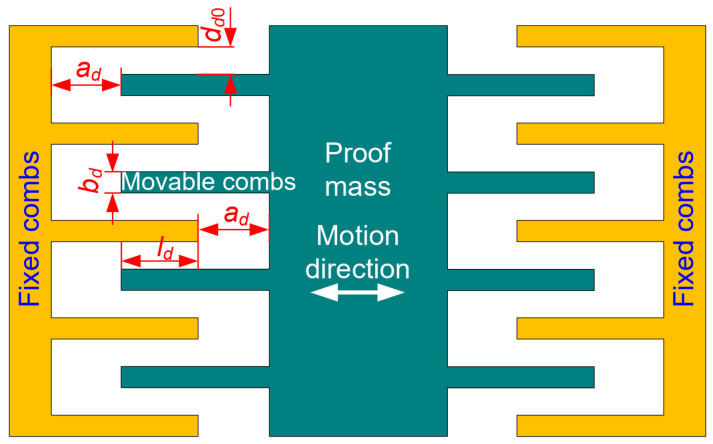
Schematic diagram of driving structure.

Area-varying capacitors are used as the electrostatic driving structure (Figure 5). When there is no applied acceleration perturbation, the movable combs, being fixedly connected to the proof mass, are located in the middle of the fixed combs. When there is an applied acceleration perturbation, the gap $d_{d0}$ remains unchanged while the overlapping length $l_{d}$ changes.

When the proof mass shifts with a displacement caused by an acceleration perturbation in the sensing direction, the differential of the drive capacitance (Figure 5) to the displacement can be expressed as:

(5) $$\frac{\partial C_{d}}{\partial y}=2n_{d}\varepsilon \frac{h_{d}}{d_{d0}}+\left(2n+1\right)\varepsilon \frac{b_{d}h_{d}}{a_{d}^{2}}$$

where $n_{d}$ denotes the number of combs, $h_{d}$ refers to the height of the combs, and $b_{d}$ denotes the width of the combs. The first term of Equation (5) is a constant while the second term varies with the changing $a_{d}$. Thus, the second term brings a nonlinearity to $\partial C_{d}/\partial y$. The ratio of the second term to the first term, i.e., the nonlinearity, is given as:

(6) $$R=\frac{\left(2n+1\right)\varepsilon \frac{b_{d}h_{d}}{a_{d}^{2}}}{2n\varepsilon \frac{h_{d}}{d_{d0}}}\approx \frac{b_{d}d_{d0}}{a_{d}^{2}}$$

**Figure 6:**
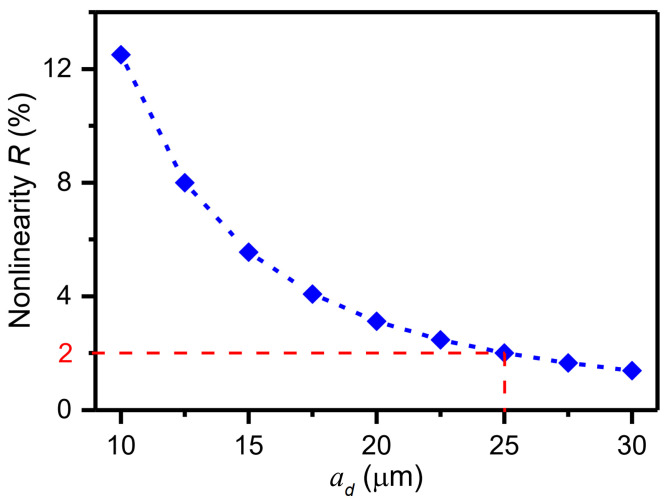
Dependence of the nonlinearity on the gap $a_{d}$.

As can be seen from Equation (6), the ratio is proportional to the comb width $b_{d}$ and the gap $d_{d0}$ and inversely proportion to gap $a_{d}^{2}$. Some parameters of the drive capacitors are constrained: $b_{d}=5$ μm; $l_{d}=40$ μm; $d_{d0}=2.5$ μm. The nonlinearity decreases while the gap $a_{d}$ increases. However, increasing the gap $a_{d}$ reduces the driving capacitance. Therefore, in this work, the nonlinearity is limited to less than 2%. The dependence of the nonlinearity on the gap $a_{d}$ is displayed in Figure 6. When the gap $a_{d}$ is greater than or equal to 25 μm, the nonlinearity R is less than 2%. As a result, the gap $a_{d}$ of 25 μm was chosen. The geometrical parameters of the designed driving structure are listed in Table 3.

#### 3.1.4. Detection Structure of the Oscillation Trajectory of the Proof Mass

**Figure 7:**
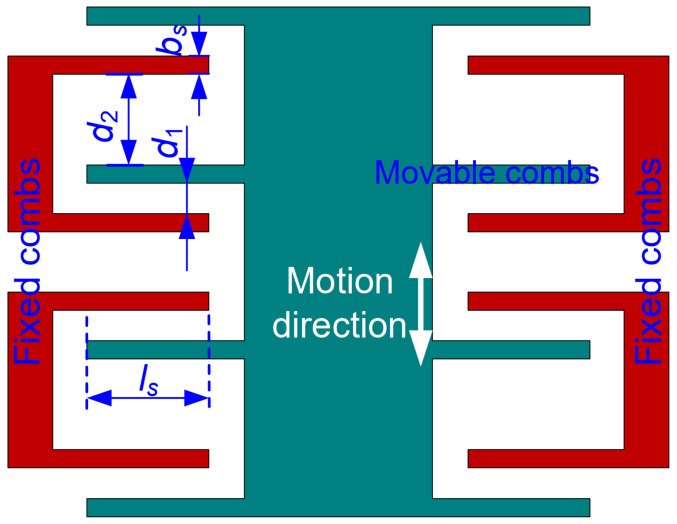
Schematic diagram of detection structure based on sense capacitors.

Gap-varying capacitors (Figure 7) are used for the detection structure of the oscillation trajectory of the proof mass. When there is no applied acceleration perturbation, the movable combs, being fixedly connected to the proof mass, are located in the middle of the fixed combs. When there is an applied acceleration perturbation, the overlapping length $l_{s}$ remains unchanged while the gaps $d_{1}$ and $d_{2}$ change. The detection structure converts the proof mass displacement into detectable capacitance, which is necessary for representing DRPs, measuring time intervals, and further calculating acceleration.

The ratio of $d_{1}$ to $d_{2}$ is defined as a variable η. Based on the parameters (Figure 7), the detection capacitance ΔC can be expressed as:

(7) $$\begin{array}{l} \Delta C=n\varepsilon lh_{s}\left(\frac{1}{d_{1}-\Delta y}+\frac{1}{d_{2}+\Delta y}\right)-n\varepsilon lh_{s}\left(\frac{1}{d_{1}+\Delta y}+\frac{1}{d_{2}-\Delta y}\right)\\ =\frac{2n\varepsilon lh_{s}}{d_{1}}\left(1-\frac{1}{\eta^{2}}\right)\left[\frac{\Delta y}{d_{1}}+{\left(\frac{\Delta y}{d_{1}}\right)}^{3}+{\left(\frac{\Delta y}{d_{1}}\right)}^{5}+\cdots \right] \end{array}$$

**Figure 8:**
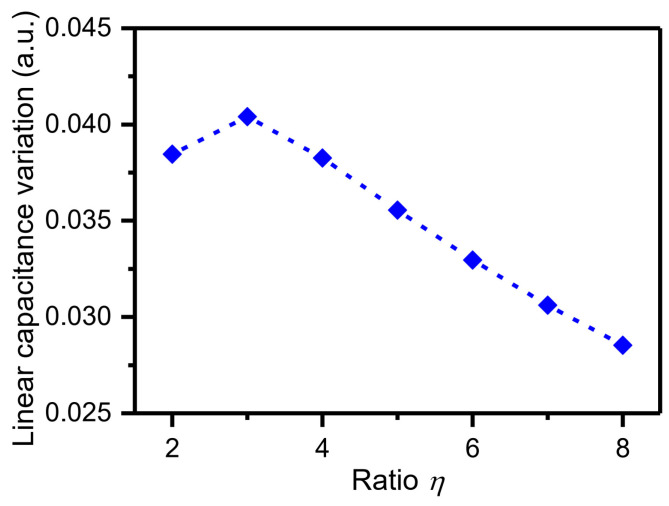
Dependence of the linear capacitance variation on the ratio η.

In the Equation (7), the part containing the first-order term of $\Delta y/d_{1}$ represents the linear capacitance variation. The other part containing the high-order terms of $\Delta y/d_{1}$ represents nonlinear capacitance variation. In this work, the nonlinearity is limited to less than 5%. Some parameters of the sense capacitors are constrained: $b_{s}=6$ μm; $l_{s}=60$ μm; $d_{1}=2.5$ μm. The dependence of the linear capacitance variation on the ratio η is displayed in Figure 8. When the ratio η is equal to 3, the linear capacitance variation reaches a maximum. Moreover, when the displacement Δy is less than 540 μm, the nonlinearity of the capacitance variation is less than 5% (Equation (7)). The resulting geometrical parameters of the designed detection structure are listed in Table 4.

### 3.2. Fabrication of Time Domain Sensor Device Using One Photomask

**Figure 9:**
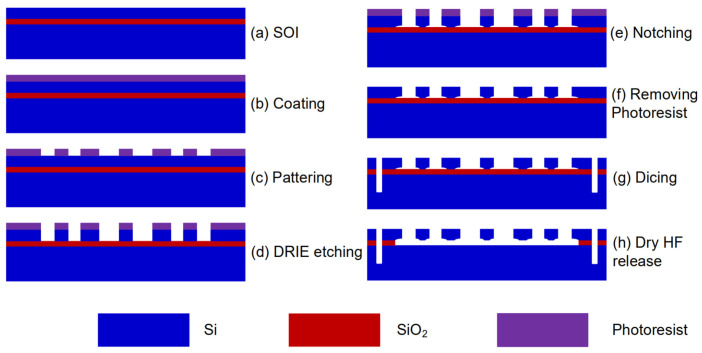
SOI fabrication process of the sensor device [23].

**Figure 10:**
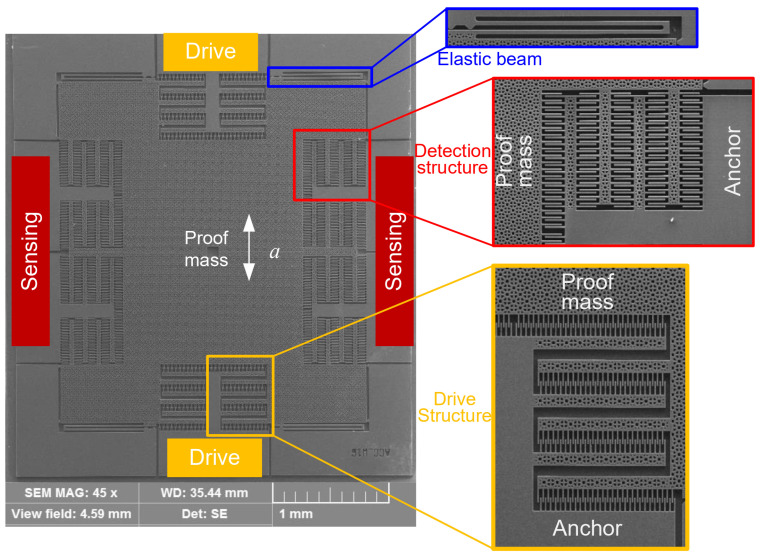
SEM image and close-up of the fabricated sensor device [23,24,30].

**Figure 11:**
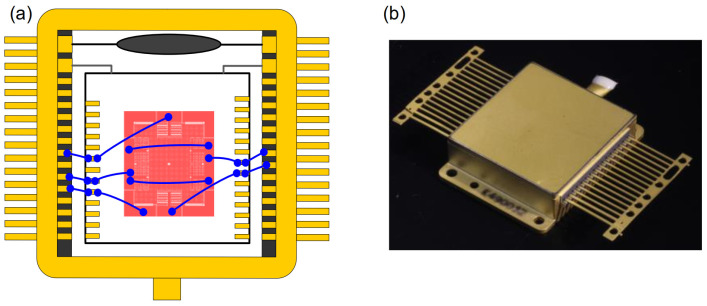
Schematic diagram of wire bonding (**a**) and the packaged device (**b**).

Based on the designed geometrical parameters described in Part 3.1, the device was fabricated using a typical silicon-on-insulator (SOI) process, as illustrated in Figure 9 [23]. The thickness of the device layer, handle layer, and oxide layer was 30, 400, and 4 µm, respectively (Figure 4a). The fabrication process involved steps such as spin coating of photoresist (b), patterning (c), deep reactive-ion etching (DRIE) (d), notching (e), removing the photoresist (f), dicing (g), and releasing the structure using dry HF (h). In the whole process, only one photomask was used. After the DRIE, the step of notching was employed for the purpose of rapidly releasing device layer. At the same time, the oxide layer below the anchors prevented over-etching. The SEM image is shown in Figure 10. The schematic diagram of wire bonding and the packaged sensor device is shown in Figure 11.

## 4. Detection Method of Developed Time Domain Acceleration Sensor

Based on the fabricated sensor device, an acceleration detection method is proposed. The detection method consists of a push–pull electrostatic drive circuit, detection circuit for the oscillation trajectory of the proof mass, and time-interval measurement based on data post-processing. The push–pull electrostatic drive is used to actuate the proof mass under a resonant state. The detection of the oscillation trajectory converts the displacement of the proof mass into a voltage. As a result, a functional relationship is built between the displacement and the voltage. The DRPs are indirectly characterized by the VRPs. The data post-processing method is used to judge the times when the proof mass passes the DRPs, extract the corresponding time intervals, and calculate the acceleration perturbation.

### 4.1. Push–Pull Electrostatic Drive Circuit

**Figure 12:**
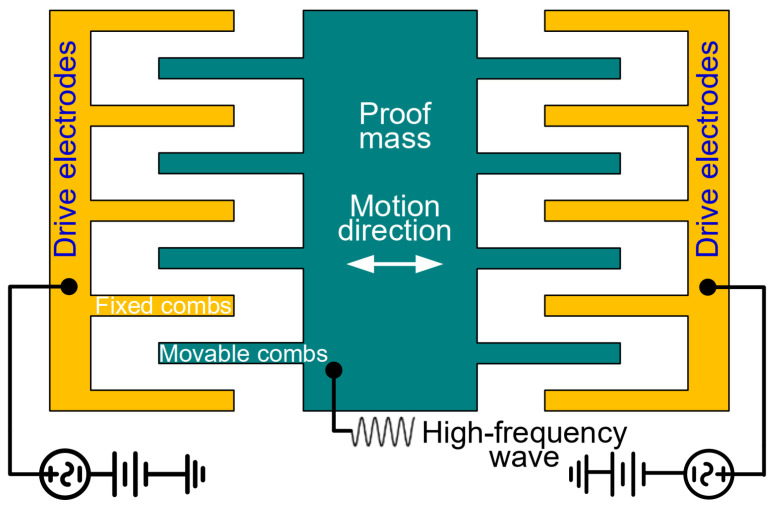
Push–pull electrostatic drive.

As shown in Figure 12, area-varying capacitors serve as differential drive electrodes. An AC drive voltage with a DC bias voltage is applied to the drive electrodes, where the DC bias voltages are equal in magnitude but opposite in direction. In addition, a high-frequency sinusoidal carrier wave is applied to the proof mass. Typically, the frequency of the carrier wave is about three orders higher than that of the AC drive signal. There is a potential difference between the movable combs fixed to the proof mass and the fixed combs connected to the drive electrodes, forming a normal electrostatic force (perpendicular to the direction of motion) and a tangential electrostatic force (along the direction of motion). The tangential electrostatic force provides the driving force while the normal electrostatic forces cancel each other. The tangential electrostatic force is given as:

(8) $$\begin{array}{l} F_{d}=\frac{1}{2}\frac{\partial C_{d}}{\partial y}{\left(V_{a}\sin\omega_{d}t+V_{d}-V_{0}\sin\omega_{m}t\right)}^{2}-\frac{1}{2}\frac{\partial C_{d}}{\partial y}{\left(V_{a}\sin\omega_{d}t-V_{d}-V_{0}\sin\omega_{m}t\right)}^{2}\\ =\frac{1}{2}\frac{\partial C_{d}}{\partial y}\left[{\left(V_{a}\sin\omega_{d}t+V_{d}\right)}^{2}-{\left(V_{a}\sin\omega_{d}t-V_{d}\right)}^{2}+2V_{0}\sin\omega_{m}t\left(-2V_{d}\right)\right]\\ =\frac{1}{2}\frac{\partial C_{d}}{\partial y}\left(4V_{a}V_{d}\sin\omega_{d}t-4V_{0}V_{d}\sin\omega_{m}t\right) \end{array}$$

where $V_{a}\sin\omega_{d}t$ denotes the AC component of the drive voltage, $V_{d}$ denotes the DC component of the drive voltage, $V_{0}\sin\omega_{m}t$ denotes the high-frequency sinusoidal carrier wave.

The frequency of the AC drive voltage is equal to the resonant frequency of the time domain sensor for the purpose of the device resonance. As mentioned above, the frequency of the carrier wave is about three orders higher than that of the AC drive voltage, and the response of the time domain sensor to the carrier wave, i.e., the second term of Equation (8), is greatly attenuated and can be neglected according to the amplitude-frequency characteristic of the second-order system. As a result, the effective electrostatic force is reduced to:

(9) $$F_{deff}=\frac{1}{2}\frac{\partial C_{d}}{\partial y}4V_{d}V_{a}\sin\omega_{d}t$$

It can be seen from Equation (9) that the magnitude of the effective electrostatic force is proportional to the differential of the drive capacitance, the DC bias voltage, and the amplitude of the AC component of the drive voltage. The frequency of the effective electrostatic force is the same as the frequency of the AC drive voltage. Correspondingly, the displacement x (i.e., the vibration amplitude A) caused by the electrostatic force is given as:

(10) $$x=A=\frac{F_{deff}}{K}Q$$

where Q refers to the quality factor of the sensor.

Submitting Equations (4), (5), (9) and (10) into Equation (3), the acceleration resolution can be rewritten as:

(11) $$\Delta a\propto \frac{\left[2n_{d}\varepsilon \frac{h_{d}}{d_{d0}}+\left(2n+1\right)\varepsilon \frac{b_{d}h_{d}}{a_{d}^{2}}\right]\sqrt{\frac{Etw^{3}W}{L_{3}w^{3}+2L_{1}^{3}W}}4V_{d}V_{a}\sin\omega_{d}t}{2m^{\frac{3}{2}}}Q\Delta t$$

It can be seen from Equation (11) that the resolution can be expressed by the structural parameters of the designed time domain accelerometer. As a result, a relationship between the acceleration resolution and the structural parameters can be built, which provides a theoretical foundation for time domain sensor device design.

### 4.2. Detection Circuit of the Oscillation Trajectory of the Proof Mass

#### 4.2.1. Representation of DRPs

For a forced vibration of a mass–spring–damper single-freedom system, when an acceleration perturbation a is applied, the center position of the oscillation trajectory of the proof mass shifts a displacement d, $d=a/\omega_{0}^{2}$ [30]. Therefore, when the acceleration perturbations, −1 g and +1 g, are applied, the shifted displacement of the center position can be expressed as:

(12) $$x_{a}=\frac{2g}{\omega_{0}^{2}}$$

**Figure 13:**
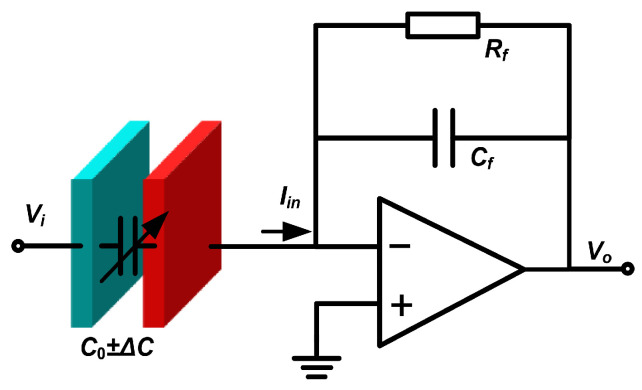
Displacement extraction based on a charge amplifier.

The shifted displacement causes a change in the spacing between the movable and fixed combs of the sense capacitors (Figure 7), which further results in a change in capacitance. Through the displacement extraction circuit based on a charge amplifier (Figure 13), the change in displacement is reflected by the change in output voltage. Notably, it is necessary carry out signal modulation and demodulation for the output voltage because the AC drive voltage is fed through to the output.

If the extracted DC voltages are $V_{-1g}$ and $V_{+1g}$ when the acceleration perturbations are −1 g and +1 g, the sensitivity of displacement to voltage is given as:

(13) $$S_{xV}=\frac{2g/\omega_{0}^{2}}{V_{-1g}-V_{+1g}}$$

Then, the functional relationship between displacement and voltage can be expressed as:

(14) $$x=\left(V-\frac{V_{-1g}+V_{1g}}{2}\right)\times S_{xV}$$

Correspondingly, the functional relationship between the DRPs and the VRPs can be expressed as:

(15) $$X_{i}=\left(V_{i}-\frac{V_{-1g}+V_{1g}}{2}\right)\times S_{xV}\;\left(i=1,2\right)$$

#### 4.2.2. Relationship between Displacement and Capacitance

**Figure 14:**
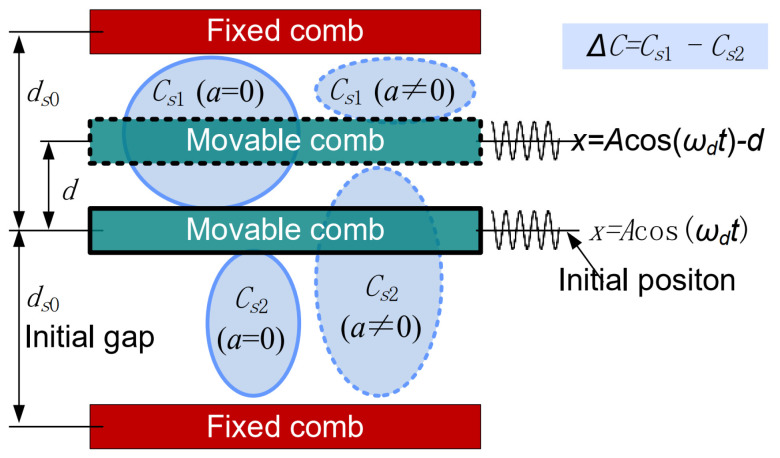
Varying sense capacitance [30].

The proof mass works under a resonant state. Thus, the movable combs of the sense capacitors fixed to the proof mass also work under a resonant state. As a result, the varying sense capacitance (Figure 14) consists of three parts: the initial capacitance $C_{s0}$ ($C_{s0}=\varepsilon_{0}l_{s}h_{s}/d_{s0}$), the capacitance change caused by acceleration perturbation $\Delta C_{sd}$ ($\Delta C_{sd}=C_{s0}d/d_{s0}$), and the capacitance change caused by the simple harmonic oscillation $\Delta C_{sv}$ ($\Delta C_{sv}=C_{s0}A\cos\omega_{d}t/d_{s0}$).

The sense capacitances $C_{s1}$ and $C_{s2}$ (Figure 14) can be expressed as:

(16) $$C_{s1}=C_{s0}+\Delta C_{sd}+\Delta C_{sv},\;C_{s2}=C_{s0}-\Delta C_{sd}-\Delta C_{sv}$$

The difference $\Delta C_{s}$ between $C_{s1}$ and $C_{s2}$ can be expressed as:

(17) $$\Delta C_{s}=C_{s1}-C_{s1}=2\left(\Delta C_{sd}+\Delta C_{sv}\right)=2C_{s0}\frac{d}{d_{s0}}+2C_{s0}\frac{A\cos\omega_{d}t}{d_{s0}}$$

It can be seen from Equation (17) that the change of differential sense capacitance is caused by the external acceleration perturbation and the harmonically oscillating proof mass.

#### 4.2.3. Differential Capacitance Detection Method of Suppressing the Same-Frequency Interference

**Figure 15:**
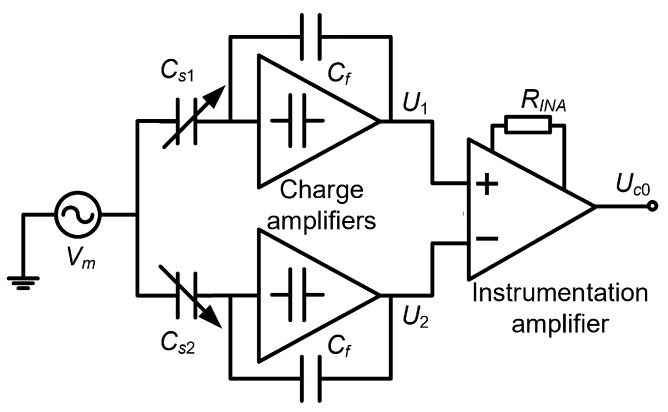
Single-carrier dual-channel integrated amplifier.

The differential capacitance $\Delta C_{s}$ consists of two terms (Equation (17)). However, only the first term corresponds to the acceleration perturbation. Consequently, the second term must be suppressed. First, the differential capacitance $\Delta C_{s}$ is converted into high-frequency AC voltage by a single-carrier dual-channel integrated amplifier (Figure 15), where the integrated amplifier consists of two identical charge amplifiers and an instrument amplifier. The commercial chips AD8065 and AD8221 were chosen to build the two charge amplifiers and the instrument amplifier, respectively. The designed sensing capacitance $C_{s1}$ and $C_{s2}$ are both equal to 1.5 pF. The feedback capacitance $C_{f}$ is set as 2.2 pF. The resistor $R_{INA}$ is set as 51 kΩ. The amplitude and frequency of the sinusoidal carrier wave are 0.5 V and 100 kHz.

**Figure 16:**
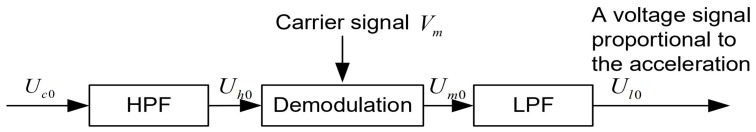
Block diagram of suppression of the same-frequency interference.

The high-frequency voltage can be expressed as:

(18) $$U_{c0}=K_{INA}{\left(U_{2}-U\right)}_{1}=K_{INA}\frac{C_{s1}-C_{s2}}{C_{f}}V_{m}=K_{INA}V_{m}\frac{\Delta C_{s}}{C_{f}}$$

where $V_{m}$ denotes carrier voltage ($V_{m}=V_{0}\sin\omega_{m}t$) and $C_{f}$ denotes feedback capacitance. The block diagram of suppression of the same-frequency interference is shown in Figure 16. Demodulation is performed on the high-frequency AC voltage $U_{c0}$ for the purpose of obtaining a voltage. The voltage is proportional to the capacitance or displacement caused by the acceleration perturbation. In addition, same-frequency interference is prevented by the high-pass filter (HPF) before the demodulation. After the demodulation, the high-frequency carrier is isolated through a low-pass filter (LPF).

The output voltages of the high-pass filter, demodulation, and low-pass filter can be expressed as:

(19) $$\left\{\begin{array}{l} U_{h0}=U_{c0}{\dot{A}}_{uh}={\dot{A}}_{uh}K_{INA}V_{m}\frac{\Delta C_{s}}{C_{f}}\\ V_{m0}=K_{m}V_{m}U_{h0}=K_{m}{\dot{A}}_{uh}K_{INA}V_{m}^{2}\frac{\Delta C_{s}}{C_{f}}=\frac{1}{2}K_{m}{\dot{A}}_{uh}K_{INA}V_{0}^{2}\frac{\Delta C_{s}}{C_{f}}\left[1-\cos\left(2\omega_{m}t\right)\right]\\ U_{l0}=\frac{1}{2}K_{m}{\dot{A}}_{uh}{\dot{A}}_{ul}K_{INA}V_{0}^{2}\frac{\Delta C_{s}}{C_{f}} \end{array}\right.$$

where ${\dot{A}}_{uh}$ denotes the amplification factor of the HPF, $K_{m}$ refers to the amplification factor of the demodulation, and ${\dot{A}}_{ul}$ denotes the amplification factor of the LPF. The output voltage $U_{l0}$ from the LPF is proportional to the capacitance or displacement caused by the acceleration perturbation.

**Figure 17:**
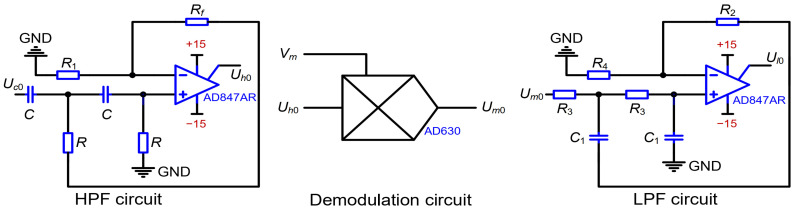
Circuits of the HPF, demodulation, and low-pass filter.

The circuits of the HPF, demodulation, and low-pass filter are shown in Figure 17. Commercial chips from Analog Devices, Inc. were used to implement the circuits. An operational amplifier AD847 was chosen for the HPF circuit. Therein, the resistors $R_{f}$, $R_{1}$, and R are equal to 1 kΩ. The capacitor C is set as 1.5 pF. As a result, the amplification factor and cut-off frequency are 2 and 234 kHz in the HPF. A demodulator AD630 was chosen for the demodulation circuit. An operational amplifier AD847 is also chosen for the LPF circuit. Therein, the resistors $R_{2}$, $R_{3}$, and $R_{4}$ are equal to 1 kΩ. The capacitor $C_{1}$ is set as 33 pF. As a result, the amplification factor and cut-off frequency in the LPF are 2 and 4.8 kHz.

### 4.3. Time Intervals Measurement Based on Data Post-Processing

**Figure 18:**
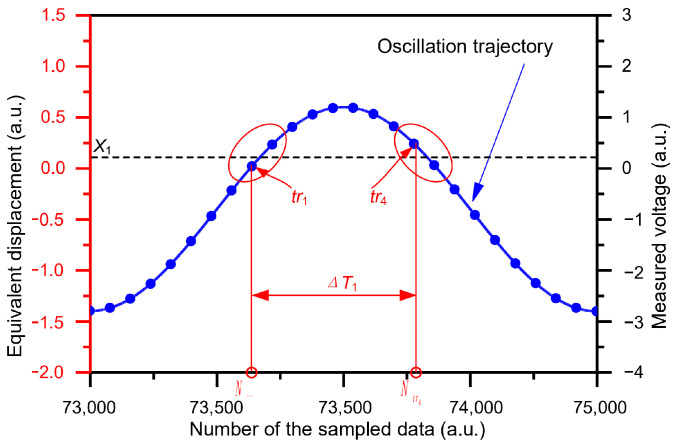
Schematic diagram of time-interval measurement [24,30].

The output voltage signal from LPF is sampled using DAQ [31]. Sampling data are post-processed using MATLAB 2012b (Figure 18). As depicted in Figure 18, the trajectory oscillation of the time domain sensor is sampled. Assuming that the sampling rate is $S_{R}$, the time-measurement accuracy is $\Delta t=1/S_{R}$. Thus, the time-measurement accuracy is tunable through varying the sampling rate. We take time interval $\Delta T_{1}$ as an example for explaining how the time intervals are measured. When DRP *X*_1_ is between two adjacent sampling data, the first datum is chosen as the trigger event. Thus, the first trigger event $tr_{1}$ and the second trigger event $tr_{4}$ are chosen in an oscillation period. The event $tr_{1}$ corresponds to sampling datum numbered $N_{tr_{1}}$ while the event $tr_{4}$ corresponds to sampling datum numbered $N_{tr_{4}}$. The difference between the two trigger events, $tr_{1}$ and $tr_{4}$, is extracted as the measured time interval $\Delta T_{1}$, i.e., $\Delta T_{1}=\left(N_{tr_{4}}-N_{tr_{1}}\right)\times \Delta t$. Utilizing the same method, the time intervals $\Delta T_{2}$ and ΔT are measured. With reference to Figure 2a and Figure 18, submitting the measured time intervals $\Delta T_{1}$, $\Delta T_{2}$, and ΔT into Equation 19, the acceleration is calculated as [24]:

(20) $$a={\left(\frac{2\pi }{\Delta T}\right)}^{2}\left[\frac{\left(X_{1}-X_{2}\right)}{\cos\left(\pi \frac{\Delta T_{1}}{\Delta T}\right)-\cos\left(\pi \frac{\Delta T_{2}}{\Delta T}\right)}\cos\left(\pi \frac{\Delta T_{1}}{\Delta T}\right)-X_{1}\right]$$

## 5. Measurement Results and Discussion

### 5.1. Dependence of Acceleration Resolution on Time-Measurement Accuracy and Vibration Amplitude

#### 5.1.1. Dependence of the Acceleration Resolution on the Time-Measurement Accuracy

**Figure 19:**
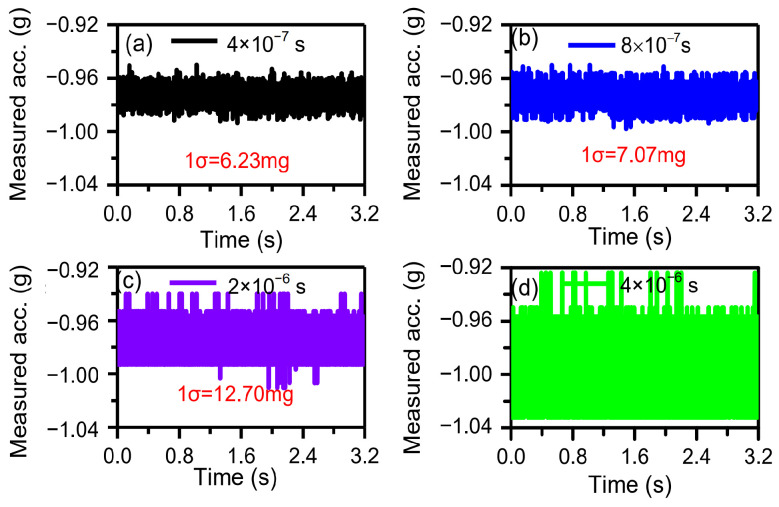
Dependence of the solved acceleration on different time-measure accuracies: (**a**) 4 × 10^−7^ s, (**b**) 8 × 10^−7^ s, (**c**) 2 × 10^−6^ s, (**d**) 4 × 10^−6^ s.

**Figure 20:**
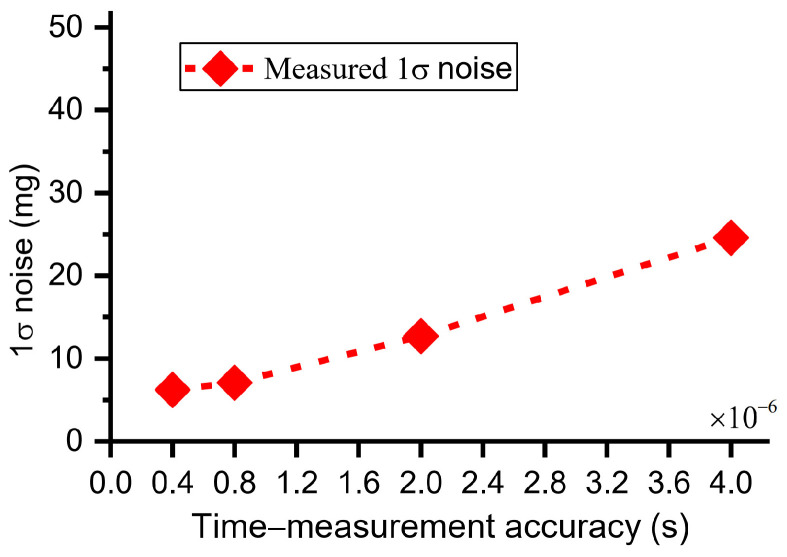
Dependence of 1σ noise on different time-measurement accuracies.

The performance of the time domain accelerometer was evaluated via the standard deviation (1σ) of the measured acceleration values [30]. Different time-measurement accuracies were achieved by sampling rate. When the sampling rates were 2.5 MHz, 1.25 MHz, 0.5 MHz, 0.25 MHz respectively, the corresponding time-measurement accuracies were 4 × 10^−7^ s, 8 × 10^−7^ s, 2 × 10^−6^ s, 4 × 10^−6^ s. The time domain sensor is mounted on dividing head to respond to external acceleration of −1 g. When the DC component of drive voltage, $V_{d}$, is 1 V and both the peak–peak amplitude and frequency of the AC component of drive voltage are 0.3 V and ~1245.88 Hz, the sensor works under resonant state with a vibration amplitude of ~342 nm and a frequency of ~1245.88 Hz. The analog signal of the sensor was sampled with different sampling rates for 3.2 s. The DRPs were set as 0 nm and 130 nm. Using the data post-processing method [30], the solved acceleration is shown in Figure 19. The 1σ noises of the solved acceleration were 6.23 mg, 7.07 mg, 12.07 mg, 24.6 mg at the time-measurement accuracies of 4 × 10^−7^ s, 8 × 10^−7^ s, 2 × 10^−6^ s, 4 × 10^−6^ s. When the time-measurement accuracies were equal to or lower than 8 × 10^−7^ s (≥8 × 10^−7^ s), the measured 1σ noises were almost proportional to the time-measurement accuracies (Figure 20). The results are consistent with the theoretical analysis (Equation (3)), which indicates that the sensor noises were mainly dependent on time-measurement accuracy. When the time-measurement accuracies were higher than or equal to 8 × 10^−7^ s (≤8 × 10^−7^ s), the measured 1σ noises were not proportional to the time-measurement accuracies. We can infer that the sensor noise was affected by not only the time-measurement accuracy but also the C–V interface circuit. Limited by the DAQ, higher time accuracies (<4 × 10^−7^ s) could not be obtained and the corresponding 1σ noises of acceleration were not measured.

#### 5.1.2. Dependence of the Acceleration Resolution on the Vibration Amplitudes

**Figure 21:**
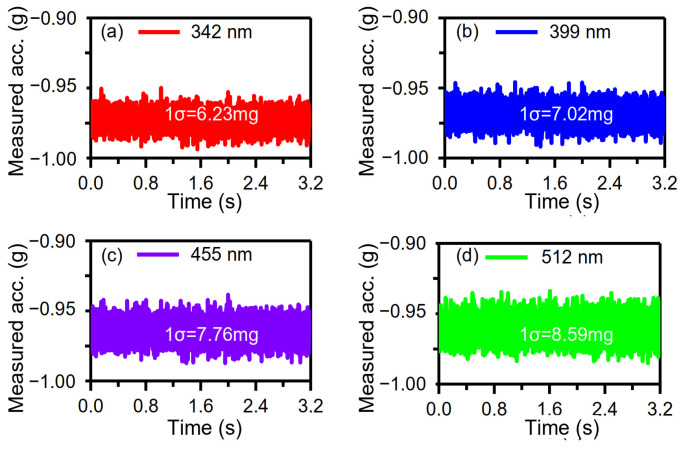
Dependence of the solved acceleration on different vibration amplitudes: (**a**) 342 nm, (**b**) 399 nm, (**c**) 455 nm, (**d**) 512 nm.

**Figure 22:**
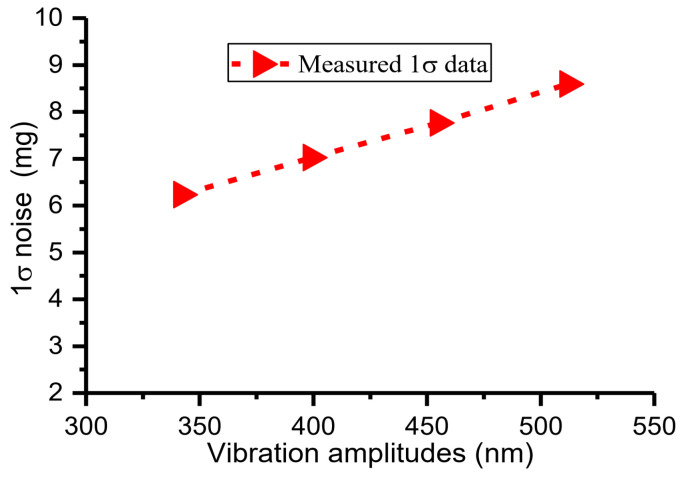
Dependence of 1σ noise on different vibration amplitudes.

The DC component of the drive voltage, $V_{d}$, remained the same, i.e., $V_{d}$ = 1 V, while the peak–peak amplitudes of the AC component of the drive voltage were set as 0.3 V, 0.35 V, 0.40 V, and 0.45 V; the driving forces varied and different vibration amplitudes of ~342 nm, ~399 nm, ~455 nm, and ~512 nm were achieved. The sensor operated in a resonant state with a frequency of ~1245.88 Hz, responding to external acceleration of −1 g. The sampling rate was 2.5 MHz and the corresponding time-measurement accuracy was 4 × 10^−7^ s. The analog signal of the sensor was sampled with different vibration amplitudes for 3.2 s. Then, the DRPs were set as 0 nm and 130 nm. The solved acceleration via data post-processing is shown in Figure 21. The 1σ noises of the solved acceleration were 6.23 mg, 7.02 mg, 7.76 mg, 8.59 mg at vibration amplitudes of ~342 nm, ~399 nm, ~455 nm, and ~512 nm (Figure 21). The measured 1σ noise was almost proportional to the vibration amplitude (Figure 22). This result is consistent with the theoretical analysis (Equation (3)).

It should be noted that the displacement and DRPs are indirectly represented by voltage and VRPs. The voltage is obtained from the varying capacitance of the sense capacitors in the C–V circuit. If the developed device rotates around the Z axis, the Coriolis force acts on the oscillating proof mass and the proof mass moves along the X direction (non-sensing direction). Then, an additional capacitance caused by the Coriolis force is coupled to the effective capacitance caused by the acceleration perturbation. Therefore, the rotation of the developed device around the Z axis affects the measured acceleration. If the displacement and DRPs of the device are physically defined as stacked tunneling electrodes [26], the trigger events are not influenced by the certain X-direction displacement caused by the Coriolis force. Therefore, the rotation of the device based on tunneling electrodes does not affect the measured acceleration. In future work, time domain sensors based on tunneling electrodes will be developed and new characteristics of the time domain sensors studied.

For the time domain sensor, the measurement range can be expressed as $a_{\max}=\omega^{2}\left(A-d_{0}\right)$ [30]. Combining this with the resolution expression $\Delta a\propto A\omega^{3}\Delta t$ (Equation (3)), we can conclude that when the vibration amplitude and resonant frequency vary, both the measurement range and resolution vary. Furthermore, when the resolution grows low, the measurement range grows large. Generally, two or more separate traditional accelerometers are required for the sensing or measurement of parameter values of significantly different amplitude, thereby increasing the cost and complexity of systems capable of wide measurement ranges. On the contrary, if a developed time domain sensor with adjustable resolution and measurement range could be utilized to sense or measure the significantly different amplitude, it would be a promising technique because the cost and complexity would be lowered.

## 6. Conclusions

A top-down design methodology and the implementation of a time domain accelerometer have been presented in this paper. The acceleration resolution of the time domain sensor can be tuned by varying the time-measurement accuracy, vibration amplitude, and resonant frequency. The desired resolution was taken as the design target. Some parameters were calculated under the constrained parameters. In this work, the time-measurement accuracy and resonant frequency were constrained, and the vibration amplitude was calculated. Then, a time domain sensor device including driving and detecting structures was detailed, designed according to the parameters, and the device was fabricated utilizing a standard SOI process. In addition, a detection method matched to the fabricated device was developed. Experimental results show that the developed time domain sensor works well, verifying the effectiveness and feasibility of the design methodology and the implementation. The acceleration resolution can be tuned by varying the time-measurement accuracies and vibration amplitudes. Representing a limitation to the current device and test platform, the resonant frequency in the acceleration resolution adjustment test cannot be tuned. In future work, a device with a special port for tuning resonant frequency will be designed for acceleration resolution enhancement and adjustment tests. The top-down design methodology can be expanded to other time domain inertial sensors, e.g., a time domain gyroscope or tilt sensor, and the results of this work can also be applied to other time domain inertial sensors.

## Figures and Tables

**Table 1:** Overall parameters of the designed time domain accelerometer.

Parameters	Value
Desired acceleration resolution	~5 mg
Resonant frequency	1.2 kHz
Maximum vibration amplitude	584 nm
Highest time-measurement accuracy	400 ns
Size	3.5 mm × 4 mm
Sensing mass	1.8 × 10^−7^ kg

**Table 2:** Geometrical parameters of the designed U-shaped elastic beam.

Parameters	Value (μm)
$L_{1}$	700
$L_{2}$	700
$L_{3}$	30
w	10
W	30
t	30

**Table 3:** Geometrical parameters of the designed driving structure.

Parameters	Value (μm)
$b_{d}$	5
$l_{d}$	40
$d_{d0}$	2.5
$a_{d}$	25

**Table 4:** Geometrical parameters of the designed detection structure.

Parameters	Value (μm)
$d_{1}$	2.5
$d_{2}$	7.5
η	3
$l_{3}$	60
$b_{s}$	6

## Data Availability

Data are contained within the article.
